# High-density contrast echocardiography for patent foramen ovale assessment: sensitivity and relationship with invasively measured shunt severity and atrial septum morphology

**DOI:** 10.1186/s44156-026-00112-8

**Published:** 2026-05-28

**Authors:** Karel Medilek, Tomas Ondrus, Tomas Mraz, Josef Bis, Jaroslav Dusek, Martin Poloczek, Martin Mates, Rudolf Praus, Marek Ballon, Juraj Hrecko, Josef Stasek

**Affiliations:** 1 First Department of Internal Medicine - Cardioangiology, University Hospital Hradec Kralove, Sokolska 585, Hradec Kralove, 50005 Czech Republic; 2 Department of Internal Medicine and Cardiology, University Hospital Brno, Jihlavska 20, Brno, 62500 Czech Republic; 3 Cardiology Department Na Homolce, University Hospital Motol and Homolka, Roentgenova 37/2, Prague, 15000 Czech Republic

**Keywords:** High density contrast, Echocardiography, Persistent foramen ovale, Shunt severity, Atrial septum morphology

## Abstract

**Background:**

Transthoracic contrast echocardiography (cTTE) is a first-line method for patent foramen ovale (PFO) assessment, but its sensitivity is reported as low and inconsistent. We aimed to investigate the sensitivity of high-density cTTE for PFO detection, and the relationship between left-heart contrast opacification grade, invasively measured shunt severity and atrial septum/PFO morphology.

**Methods:**

Fifty-five consecutive patients with proven PFO on contrast transoesophageal echocardiography (cTOE) underwent cTTE with high density contrast (colloid, mannitol) and invasive quantification of right-to-left shunt severity using the thermodilution method. Echocardiographic shunt severity was classified according to the degree of left atrial (cTOE) or left atrial/ventricular (cTTE) opacification as grade 0 (no bubbles), grade 1(mild), grade 2 (moderate) grade 3 (significant opacification).

**Results:**

For PFO detection, the sensitivity of rest + Valsalva cTOE was 100%. Compared with this reference, sensitivity of rest + Valsalva cTTE was 98.2%, Valsalva cTTE 97.7%, rest cTTE, 83.9% and rest cTOE, 77.3%. No significant association was observed between left-heart opacification grade on rest or Valsalva cTTE/cTOE and invasively measured right-to-left shunt severity categorized as < 10%, 10–19%, or ≥ 20% (*p* = 0.10–0.26). A moderate association only was identified between rest + Valsalva cTOE left atrium opacification grade and < 10% and ≥ 10% right-to-left shunt severity (*p* = 0.024, Cramér’s coefficient = 0.368). No significant relationship was found between left-heart opacification grade and the presence of atrial septal aneurysm, Eustachian valve/Chiari’s network, or PFO channel width and length (*p* = 0.45–0.77). Compared with rest cTOE, rest cTTE demonstrated equal contrast opacification grade in 56% and higher in 40%. Valsalva cTTE showed equal contrast opacification grade in 29% and higher in 46% of studies vs. Valsalva cTOE.

**Conclusions:**

In studies using high-density contrast, cTOE and cTTE with Valsalva, but not rest studies, are highly sensitive for PFO assessment. There is no clinically relevant relationship between the left-heart bubble opacification grade and invasively measured right-to-left shunt severity or atrial septum morphology. The left-heart contrast opacification grade is equal to or higher in cTTE than in cTOE, especially in rest studies.

## Background

Patent foramen ovale (PFO) is a flap-like opening resulting from incomplete fusion of the septum primum and septum secundum at the fossa ovalis. Its overall reported prevalence on autopsy is 27%, with a positive relationship between PFO and cryptogenic stroke (CS) observed in up to 46% of individuals < 55 years of age and 21% in those > 55 years [1, 2]. In the RESPECT trial, closure of a PFO was associated with a lower rate of recurrent ischaemic strokes than medical therapy alone, providing evidence supporting PFO closure in selected patients with CS [3].

The contrast echocardiography effect was first described in 1968 in an experimental in vitro model using agitated saline, followed shortly by studies using 5% dextrose and agitated patient’s blood [4–6]. Twelve years later, an experimental study confirmed that echo reflection in a contrast study was primarily due to microbubbles formed during mixing of these agents with gas [7]. The non-transpulmonary contrast agents currently used for PFO detection contain microbubbles that are generally too large to pass through the pulmonary circulation [8, 9].

Contrast transcranial doppler (cTCD), contrast transoesophageal (cTOE), and contrast transthoracic (cTTE) echocardiography represent the recommended methods for right-to-left intracardiac shunt assessment. In most cases, precise PFO diagnosis requires a combination of techniques, with cTCD or cTTE recommended as the first-line method [10]. Due to technical advancements, the sensitivity of cTTE has improved over the last 30 years from 51% to 80% [11]. According to meta-analyses cited in the latest European Society of Cardiology PFO statement and European Stroke Organisation guidelines, the reported sensitivity of cTTE compared with cTOE is 88% and 71%, respectively [10, 12]. In the latter meta-analysis, colloid contrast was used in 4 of 20 studies, all of them published more than 20 years ago, with reported sensitivity ranging from 13 to 61% [10]. However, some studies suggest superior sensitivity of colloid contrast compared with normal saline [13, 14]. Additionally, the diagnostic performance of cTOE may improve with administration of larger contrast volumes and multiple boluses [15, 16].

The risk of a PFO being a causative pathology in patients with CS is usually linked to the left-heart opacification degree, PFO tunnel width and length, presence of interatrial aneurysm or hypermobile septum, and Chiari’s network or a prominent Eustachian valve [12, 17–19]. A severe shunt is usually defined by the appearance of more than 20–30 microbubbles in the left atrium within three cardiac cycles after right atrial opacification [20, 21]. The interplay between left-heart bubble contrast opacification degree and invasively measured shunt severity or PFO morphology has not been clinically studied, except in an experimental fluid dynamics model assessing the impact of PFO tunnel geometry on PFO flow behaviour [22].

We aimed to investigate the relationship between left-heart contrast opacification degree using a high-density non-transpulmonary contrast agent, invasively measured shunt severity, and atrial septum morphology, and to assess the sensitivity of cTTE using high-density contrast with and without Valsalva manoeuvre for PFO detection.

## Methods

### Study population

The MEASURE-PFO study [23] included 151 consecutive patients with CS or systemic embolisation and PFO proven by cTOE in six tertiary centres in the Czech Republic. All patients underwent invasive measurement of the right-to-left shunt followed by successful PFO closure. For contrast echocardiography studies, high-density solutions (gelatine, mannitol), dextrose or normal saline (without blood) were used. The indication for PFO assessment was at the discretion of the referring neurologist. All patients provided written informed consent. In the present sub study, we enrolled 55 patients with CS from three cardiac centres in whom high-density contrast was used and cTTE was available.

### Cardiac catheterisation

Femoral access was used for the catheterisation procedure. The pressure in the aorta was measured using the Sensis^®^ hemodynamic system (Siemens Healthineers AG, Erlangen, Germany). Systemic blood flow, pulmonary blood flow and right-to-left (R-L) shunt were determined using the thermodilution Inntherm^®^ system at rest and during Valsalva manoeuvre, if necessary facilitated by assistant applying manual pressure to the patient’s abdomen. At rest, 10 ml of ice-cold saline was injected into the inferior vena cava near its opening into the right atrium. During the Valsalva manoeuvre, the systemic pressure was monitored, and at the time of the short pressure drop to about 50% of the initial systemic pressure, the manoeuvre was stopped and the saline was injected. The temperature of the systemic blood was measured by a thermal probe positioned in the descending aorta just below the aortic arch. The presence and quantification of the R-L shunt were determined by the blood temperature change in the descending aorta, as previously described [23].

### Echocardiography

cTOE and cTTE were performed prior to PFO closure using Vivid E95 (GE^®^) with 6Vc-D TTE and 6CT-D TOE probes and EPIQ CVXi or CX50 with X5-1 or X51c TTE and X7-2t or X8-2t TOE probes (Philips^®^). All patients provided written informed consent for TOE. Peripheral venous access (≥ 20G) was preferably obtained in the cubital vein, without side preference.

#### Contrast transthoracic echocardiography

A standard TTE protocol was performed according to recommendations [24, 25]. For the contrast study, retrospective ECG-gated acquisition was selected with loops of at least ten RR intervals. 19 mL of Gelaspan^®^ 4% (B Braun) or Mannitol 20% (Fresenius Kabi) plus 1 mL of air were agitated between two 20 ml luer lock syringes connected via a three-way stopcock until a white suspension was obtained. A rapid contrast bolus was injected into a straight intravenous line immediately after preparation for the rest study. At least 5 RR intervals of the whole 10 RR loop were acquired following right atrial opacification. If no contrast was observed in the left heart, the test was repeated. In the next step, patients were trained to perform an effective Valsalva manoeuvre by taking in a deep breath, closing their mouth, and “bearing down” forcefully against the echocardiographer’s fist pushing to the patient’s abdominal wall, followed by abrupt strain release. Afterwards, patients were asked to make a Valsalva manoeuvre with the same force but without assistance. The operator performing TTE made sure to keep all four chambers in the apical four-chamber view visible during the Valsalva strain and release phases. This was practised several times. The contrast was quickly injected at the initiation of the Valsalva manoeuvre, which was abruptly released after complete administration of the 20 mL contrast, at the time of its appearance in/opacification of the right atrium (usually 7–8 s after initialisation of the Valsalva manoeuvre). If no left-sided contrast was observed or image quality was compromised by translational movement, the test was repeated.

#### Contrast transoesophageal echocardiography

Topical pharyngeal anaesthesia using lidocaine spray was administered. A standard TOE protocol was followed [26, 27]. When accomplished, the interatrial septum was visualized using the mid-oesophageal short-axis 40–60° view and the rest contrast study mirroring cTTE was performed. If negative, the test was repeated in the bi-caval view. Next, the Valsalva study was performed in the same way as in cTTE in short axis 40–60° view. If negative, it was repeated in bi-caval view and also repeated if necessary.

A positive study was defined as the presence of contrast in the left atrium (cTOE) or the left heart (cTTE) within three cycles after the right heart opacification (the rest study)/strain release (Valsalva study). Contrast coming into the left heart later was not considered as a positive study. Passage of the contrast through the interatrial septum channel on cTOE had to be directly visualized to confirm PFO presence. If this was not the case, every effort was made to localize the site of the shunt (multiperforated PFO etc.) and to rule out contrast inflow from the pulmonary vein.

**Fig. 1:**
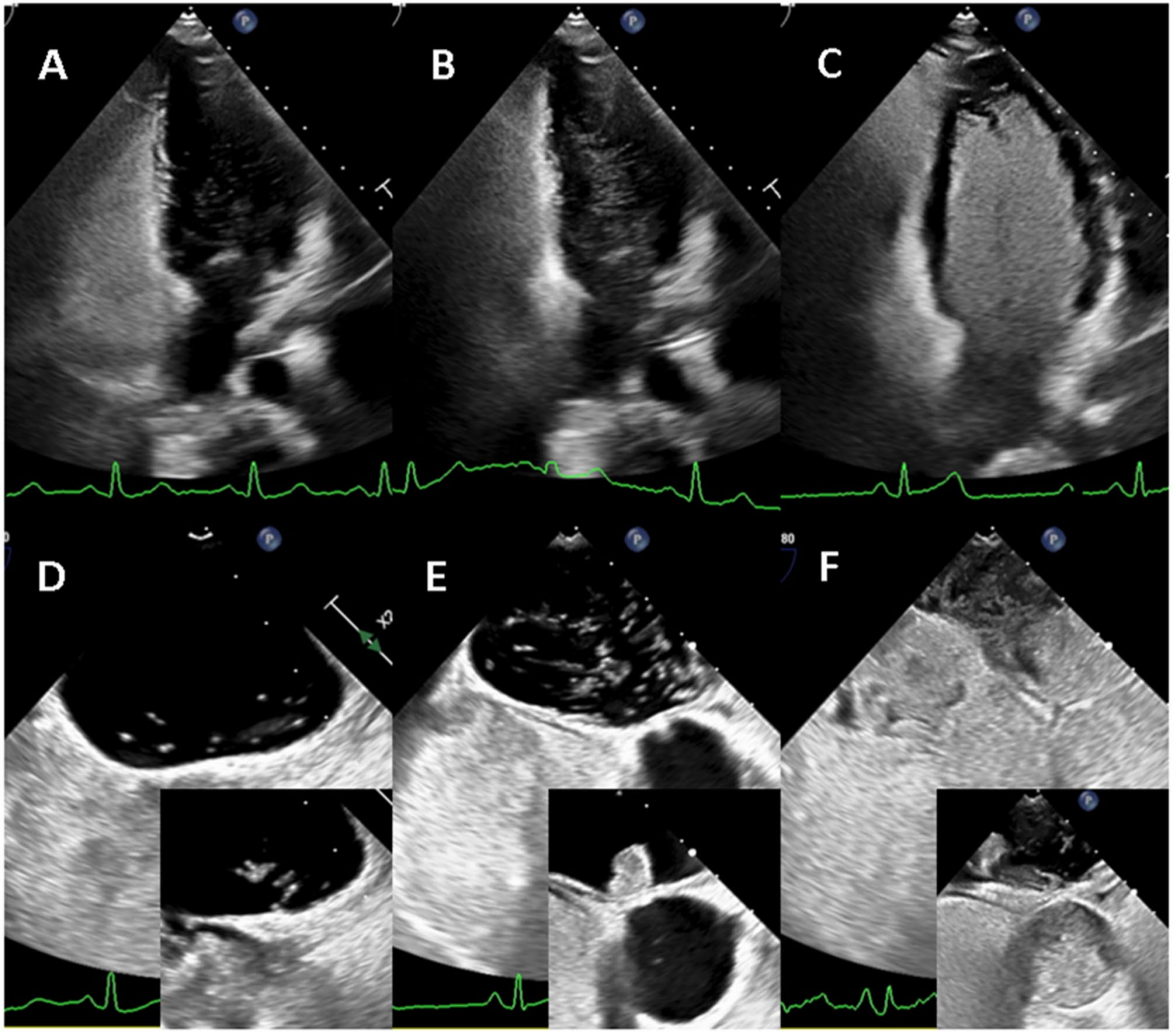
Left heart contrast opacification grading. **A** - cTTE grade 1, **B** cTTE grade 2, **C** - cTTE grade 3, **D** - cTOE grade 1, **E** - cTOE grade 2, **F** - cTOE grade 3. Small images in D-E document visualisation of the contrast passing through the PFO before the left heart opacification in cTOE. Gelaspan 4% 20 ml bolus

The left heart contrast opacification was graded as grade 0: no bubbles, grade 1: mild, grade 2: moderate and grade 3: severe left atrium contrast opacification in cTOE and left atrium/ventricle opacification in cTTE (Fig. 1). A single frame showing the maximum contrast opacification of the left atrium (cTOE) or left heart (cTTE) captured within three cycles after right heart opacification in the rest study, or after strain release in the Valsalva study, was selected for grading.

The length and width of the PFO channel at rest in the mid-oesophageal short-axis 40–60° and bi-caval projections were measured. The larger diameter was considered. The presence of the Eustachian valve or Chiari’s network was recorded; atrial septal aneurysm was defined as a 10 mm bulging of the septum tissue to the left/right side. The aim was to avoid sedation to ensure a good-quality Valsalva manoeuvre during cTOE, confirmed by the presence of atrial shifting to the left. If needed, a minimum dose of midazolam allowing completion of the whole cTOE with reliable Valsalva manoeuvre was titrated in 1 mg increments. All studies were analysed in a core lab by an experienced echocardiographer holding EACVI accreditation in adult TTE, with nearly twenty years of experience in PFO contrast studies.

### Statistical analysis

Continuous variables were summarised as means with standard deviations (SD), while categorical variables were presented as frequencies and percentages. The normality of continuous data distributions was assessed using the Shapiro–Wilk test. For comparisons across multiple groups, ANOVA on ranks (Kruskal-Wallis test) was applied when normality assumptions were not met, followed by Dunn’s post hoc test with Bonferroni correction. Categorical variables were evaluated using the chi-squared test, with Fisher’s exact test applied when expected frequencies were below 5. Cramér’s V was used to assess the strength of the association between categorical variables. Agreement between cTTE and cTOE for left heart opacification was assessed using Kendall’s τ coefficient. Systematic differences between cTTE and cTOE were evaluated using McNemar’s test. Statistical significance was set at *p* < 0.05. All statistical analyses were performed using SPSS Statistics software, version 26 (IBM Corp., Armonk, NY, USA). Graphical analyses were performed using SigmaPlot, version 14.

## Results

Fifty-five patients who met the inclusion criteria were enrolled in the study. Their baseline characteristics are summarised in Table 1.

**Table 1:** Baseline characteristics of the studied group

age (years)	*n* (% *N*) / mean ± SD
	44.9 ± (7.6)
gender	
male	38 (69.1%)
female	17 (30.9%)
BMI	28.3 ± 5.1
BSA	2.1 ± 0.2
creatinine (mmol/l)	78.3 ± 14.2
haemoglobin (g/l)	143 ± 14
height (cm)	178 ± 9
weight (kg)	89.9 ± 18
asthma bronchiale or COPD	3 (5.5%)
diabetes	3 (5.5%)
hyperlipidaemia	26 (47.3%)
hypertension	16 (29.1%)
contrast site injection	
peripheral line	53 (96.3%)
inferior vena cava	2 (3.7%)
type of contrast	
Gelaspan 4%	37 (67%)
Mannitol 20%	18 (33%)
sedation during cTOE	12 (18.5%)
sedation dose (ml)	2.6 ± 0.7
Chiari network/Eustachian valve	24 (43.6%)
PFO channel length (mm)	13.2 ± 4.7
PFO channel width (mm)	2.9 ± 2.0
atrial septum aneurysm	29 (52.7%)

BMI - body mass index, BSA - body surface area, COPD - chronic obstructive pulmonary disease

Sensitivity of rest + Valsalva cTOE was 100%, as all patients underwent successful PFO occlusion. cTTE with Valsalva manoeuvre was found to be highly sensitive for PFO detection. In contrast, rest cTTE and cTOE studies showed low sensitivity (Table 2).

**Table 2:** Sensitivity of high-density bubble studies for PFO detection

method A	method B	sensitivity A vs. B
cTTE rest + Valsalva	cTOE rest + Valsalva	98.2%
cTTE Valsalva	cTOE rest + Valsalva	97.7%
cTTE rest	cTOE rest + Valsalva	83.9%
cTOE rest	cTOE rest + Valsalva	77.3%
cTTE Valsalva	cTOE Valsalva	97.6%
cTTE rest	cTOE rest	83.6%

No association was observed between invasively measured shunt severity (0%, < 10%, 10–19%, ≥ 20%) and left heart contrast opacification grade in either resting or Valsalva cTTE and cTOE studies. (Table 3). When the shunt severity was categorised as < 10% and ≥ 10%, a moderate association only was revealed between the rest + Valsalva studies opacification grade and shunt severity in the cTOE (*p* = 0.024, Cramér’s coefficient 0.368), but not in the cTTE group (Table 4).

**Table 3:** Shunt severity vs. left heart contrast opacification grade for the rest and Valsalva cTTE/cTOE

	severity of R-L shunt				*p*
	not detected	< 10%	10–19%	≥ 20%	
**left heart opacification rest cTTE*** n* = 55					0.16
Grade 0: no bubbles	3 (50.0%)	4 (19.0%)	1 (6,7%)	1 (7.7%)	
Grade 1: mild opacification	1 (16.7%)	9 (42.9%)	4 (26.7%)	4 (30.8%)	
Grade 2: moderate opacification	0 (0.0%)	7 (33,3%)	6 (40.0%)	5 (38.5%)	
Grade 3: significant opacification	2 (33.3%)	1 (4,8%)	4 (26.7%)	3 (23.1%)	
**left heart opacification Valsalva cTTE*** n* = 44					0.20
Grade 0: no bubbles	1 (25.0%)	2 (10.5%)	0 (0.0%)	1 (11.1%)	
Grade 1: mild opacification	1 (25.0%)	1 (5.3%)	1 (8.3%)	0 (0.0%)	
Grade 2: moderate opacification	0 (0.0%)	10 (52.6%)	2 (16.7%)	4 (44.4%)	
Grade 3: significant opacification	2 (50.0%)	6 (31.6%)	9 (75.0%)	4 (44.4%)	
**left atrium opacification rest cTOE*** n* = 55					0.10
Grade 0: no bubbles	2 (33.3%)	6 (28.6%)	1 (6,7%)	2 (15.4%)	
Grade 1: mild opacification	2 (33.3%)	9 (42.9%)	6 (40.0%)	7 (53.8%)	
Grade 2: moderate opacification	0 (0.0%)	6 (28.6%)	6 (40.0%)	4 (30.5%)	
Grade 3: significant opacification	2 (33.3%)	0 (0.0%)	2 (13.3%)	0 (0.0%)	
**left atrium opacification Valsalva cTOE*** n* = 41					0.26
Grade 0: no bubbles	0 (0.0%)	0 (0.0%)	0 (0.0%)	0 (0.0%)	
Grade 1: mild opacification	2 (50.0%)	9 (50.0%)	1 (10.0%)	2 (22.2%)	
Grade 2: moderate opacification	2 (50.0%)	7 (38.9%)	5 (50.0%)	5 (55.6%)	
Grade 3: significant opacification	0 (0.0%)	2 (11.1%)	4 (40.0%)	2 (22.2%)	

Chi-squared test. R-L - right to left

**Table 4:** < 10% and ≥ 10% shunt severity vs. left heart contrast opacification grade for the rest + Valsalva cTTE/cTOE

	severity of R-L shunt		*p*
	< 10%	≥ 10%	
**left heart opacification rest + Valsalva cTTE**			0.206
Grade 0: no bubbles	1 (3.7%)		
Grade 1: mild opacification	4 (14.8%)	1 (3.6%)	
Grade 2: moderate opacification	12 (44.5%)	11 (39.3%)	
Grade 3: significant opacification	10 (37.0%)	16 (57.1%)	
**left atrium opacification rest + Valsalva cTOE**			0.024
Grade 1: mild opacification	11 (40.7%)	3 (10.7%)	
Grade 2: moderate opacification	12 (44.4%)	15 (53.6%)	
Grade 3: significant opacification	4 (14.8%)	10 (35.7%)	

Chi-squared test, R-L - right to left

No significant association was also found between left heart opacification grade and the presence of an atrial septal aneurysm, Eustachian valve/Chiari´s network, or PFO width and length. (Table 5).

**Table 5:** Relationship between atrial septal morphology and the left heart opacification grade, p value

	anatomical interatrial septum/PFO features			
	PFO width	PFO length	Chiari´s network/ Eustachian valve	aneurysm
left heart opacification grade rest cTTE	0.17	0.49	0.10	0.45
left heart opacification grade Valsalva cTTE	0.37	0.69	0.47	0.73
left atrium opacification grade rest cTOE	0.55	0.16	0.64	0.46
left atrium opacification grade Valsalva cTOE	0.81	0.43	0.29	0.77

Chi-squared and Kruskal-Wallis tests

Finally, the left heart opacification grade between cTTE and cTOE was compared. In rest studies, cTTE produced the same degree of opacification as cTOE in 55.5% of cases and a higher degree in 40%, resulting in similar or superior performance in 95.5% of examinations. A moderate and statistically significant correlation and difference were observed between cTTE and cTOE opacification grades (Kendall’s τ = 0.53, *p* < 0.001 and χ² = 26.83, *p* = 0.002). In Valsalva studies, the left heart opacification grade on cTTE matched that of cTOE in 29% of cases and exceeded it in 45.5%, indicating a comparable or higher opacification degree in 74.5% of cases. However, both the correlation and the difference between cTTE and cTOE opacification grades were weak and not statistically significant (Kendall’s τ = 0.14, *p* = 0.23 and χ² = 13.23, *p* = 0.15).

## Discussion

This study on high-density contrast echocardiography in investigation of the presence of PFO in CS patients demonstrated: (i) high sensitivity of Valsalva cTOE and cTTE for PFO detection, (ii) no clinically relevant relationship between the left heart opacification grade and invasively measured left-to-right heart shunt severity, (iii) no significant association between the left heart opacification grade and anatomical features of the interatrial septum/PFO, (iv) equal or higher left heart opacification grade in cTTE vs. cTOE, especially in rest studies.

In two studies with 91 patients included in the cTOE meta-analysis from Mojadidi, reflecting the usual practice (i.e., contrast injection into the peripheral line and Valsalva manoeuvre performance), the sensitivity of cTOE compared with autopsy, cardiac surgery, and/or catheterization (defined as successful catheter passage through the PFO) as the reference was 89–91% [28]. Furthermore, cTOE studies demonstrated a significantly lower prevalence of PFO (13%; 95% CI: 8–18%) compared with autopsy studies (25%; 95% CI: 20–29%; *p* = 0.004) [12]. The imperfect diagnostic accuracy of cTOE is commonly attributed to patient intolerance of the TOE probe or suboptimal Valsalva manoeuvre performance during sedation, which may result in up to 50% of PFOs being missed [29, 30]. Notably, sedation use during cTOE is frequently unreported in published studies [31–33]. In addition, the sensitivity of PFO detection is influenced by the number of contrast injections performed during Valsalva cTOE studies [16]. Despite these limitations, cTOE is considered the gold standard for PFO detection [12]. In our study, patients were specifically trained in proper Valsalva manoeuvre execution, and strict operator adherence to correct technique was required. Visualisation of the contrast passing through the PFO channel on cTOE was mandatory for PFO diagnosis, and high-density contrast was used. Sedation was required in < 20% of patients. These methodological factors likely contributed to the observed 100% sensitivity of cTOE compared with successful PFO occlusion in our study, highlighting the importance of an experienced imaging specialist performing cTOE for PFO assessment [34].

**Fig. 2:**
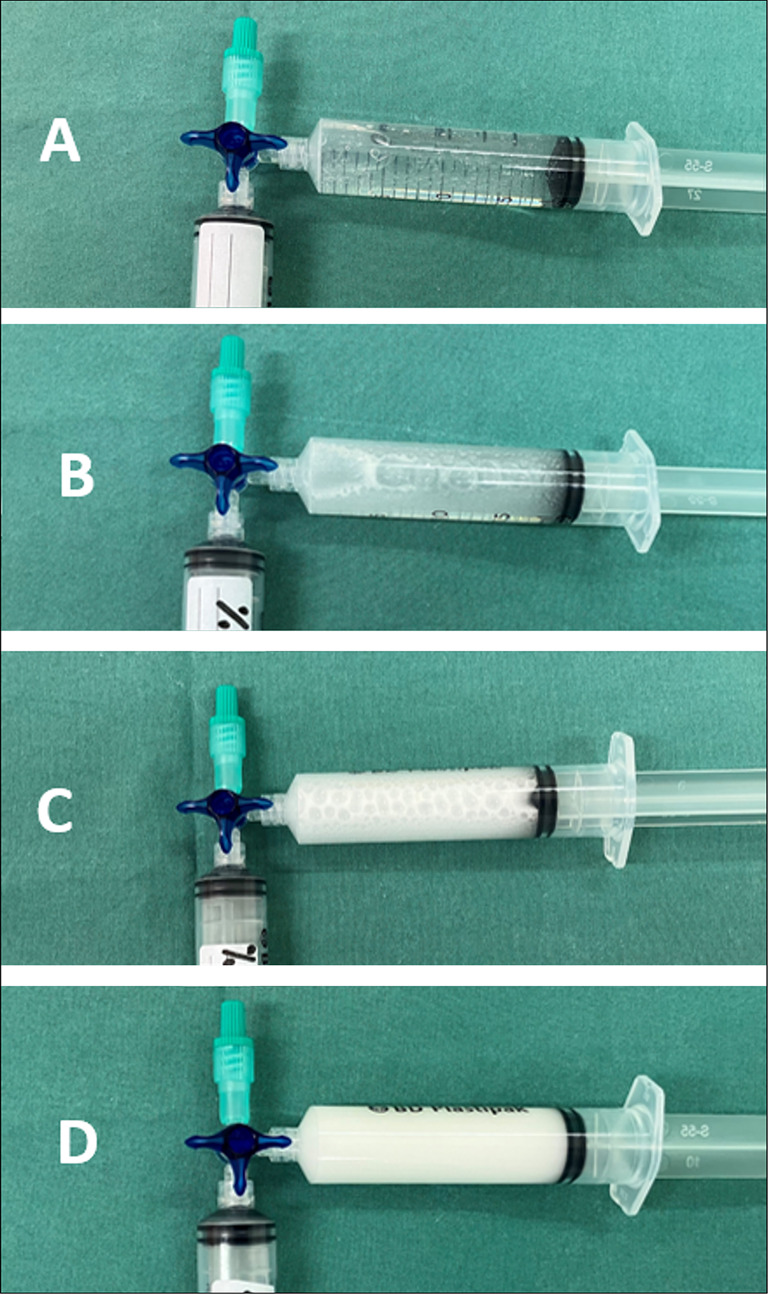
Contrast density immediately after agitation. **A** - normal saline, **B** − 10% dextrose, **C** – Mannitol 20%, **D** - Gelaspan 4%

There is heterogeneity in the type of contrast and Valsalva technique use in the published studies comparing cTTE to cTOE; saline with or without blood prevailing in 80% and the contrast injected during the provocative manoeuvre in 66% [10, 35]. The reported sensitivity of saline-based cTTE for PFO detection ranges from 23% to 92%, while colloid-based contrast demonstrates sensitivities between 30% and 61% [36]. Second harmonic imaging improved diagnostic cTTE performance, with sensitivity of 91%, comparable to the 96% sensitivity attributed to cTCD [35, 37]. In our study, we used high-density contrast agents, primarily gelatine, achieving excellent Valsalva cTTE sensitivity for PFO detection > 95%. One of the reasons, according to our experience, is better gelatine bubble stability and opacification of the right and left heart during cTTE/cTOE in comparison with normal saline contrast (Figs. 1 and 2). This is supported by studies demonstrating higher sensitivity of PFO detection by gelatine solution vs. normal saline contrast. Gelatine exhibits higher density and stability of microbubbles due to lower surface tension and longer duration of the contrast presence in the right heart than normal saline. The air–gelatine mixture preparation at the bedside is fast; the contrast is stable already after three shakes through the stopcock [9, 13, 14]. Adding blood to normal saline stabilises microbubbles, increases their number, and makes them smaller than in normal saline alone [38–40]. This increases the contrast stability and detection time in vitro and in vivo, and improves the detection of right-to-left shunt [41–43]. Behaviour of the gelatine and saline with blood seems to be, therefore, similar. Indeed, our initial clinical experience shows that the visual density of the right heart opacification using Gelaspan 4% and normal saline with blood does not differ significantly. However, there are no published head-to-head studies comparing the accuracy of the saline with blood and colloid solution for PFO detection.

The use of modified gelatine appears to be safe, a potential risk of anaphylaxis associated with its use is low, although data from intensive care settings remain conflicting [44, 45]. We found only one published case report on anaphylaxis during contrast echocardiography [46]. An association has been identified between allergic reactions to red meat and sensitisation to gelatine and galactose-α-1,3-galactose (α-Gal) [47]. As a precaution, we avoid using gelatine in patients with a known history of severe allergic reactions. From a practical perspective, gelatine is also easier and more hygienic to handle than mixing normal saline with blood, particularly with regard to infection control. For these reasons, gelatine-based contrast agents have been the first-line choice in our echocardiography laboratory for more than two decades.

In a recently published study by Takaya, normal saline cTTE was superior to cTOE for predicting large left-to-right shunts (79% vs. 28% of patients) [21]. One possible explanation may relate to our findings demonstrating better opacification of the left heart with cTTE compared with cTOE, although a different contrast agent was used (Fig. 1) Additionally, in cTTE, the entire left heart is visualised in apical four chamber view, by contrast to the cTOE study, which is focused on the presence of the contrast agent in a 2D often foreshortened image of the left atrium.

The risk of PFO-associated cryptogenic stroke (CS) is commonly assessed based on the size of right-to-left shunting, as estimated by the left heart contrast degree, and the presence of high-risk anatomical features such as PFO width, atrial septum aneurysm (ASA), septal hypermobility, and a prominent Eustachian valve. Conceptually, a large PFO may facilitate greater right-to-left shunting, a mobile ASA may promote intermittent opening of the PFO during the cardiac cycle, and a prominent Eustachian valve may direct thrombi from the inferior vena cava toward the PFO. Indeed, in a metanalysis from Overell, the presence of ASA was associated with a higher risk of ischaemic stroke in comparison to healthy subjects (OR 2.35). Individual observational studies reported even higher risk (OR 3.65) [48, 49]. However, we found no association between the left heart contrast opacification grade and the presence of high-risk PFO features. This may be explained by the high prevalence of ASA (> 50%) in our study population, reflecting the selected cohort of high-risk patients in whom additional risk stratification based solely on anatomical features may be challenging.

Scoring systems for high-risk PFO have been developed and include PFO channel width (≥ 2 mm) and length (≥ 10 mm), the presence of ASA or hypermobile interatrial septum, a prominent Eustachian/Chiari’s valve, straddling interatrial thrombus, concomitant pulmonary embolism or deep vein thrombosis, and a large shunt at rest and during the Valsalva manoeuvre (> 20 microbubbles in the left atrium) [50]. Our study is the first to compare the left heart contrast opacification grade and invasively measured shunt severity, showing no relevant relationship, apart from a moderate association of the left atrium contrast grade on rest + Valsalva cTOE studies with larger shunts > 10%. Notably, some patients had no invasively measurable shunt despite evidence of a right-to-left shunt on cTTE/cTOE (Table 3) This can be explained by relative hypovolaemia, which may have been present in some patients despite instructions not to refrain from clear fluids before undergoing invasive investigations. Additionally, some patients may have had difficulty performing an adequate Valsalva manoeuvre during invasive measurements, as manual pressure was applied to the femoral access site by the operator to prevent bleeding, which may have caused discomfort and limited the Valsalva manoeuvre quality. However, appropriate Valsalva manoeuvre performance was monitored invasively by the pressure curve changes.

Furthermore, the recommended approach of counting microbubbles in the left atrium within three cardiac cycles after the right heart opacification is impractical in cases of high-density contrast. The left atrium often becomes densely and homogeneously opacified, making individual bubble counting unreliable. Bubbles do not necessarily travel linearly across the left atrium, and the same bubbles may persist across multiple cardiac cycles, leading to potential overcounting. Conversely, the left atrial size on tomographic TOE slices is frequently foreshortened, so some bubbles are inevitably missed. Consistent with these challenges, a recent paper by Su et al. reported that quantified, guideline-recommended ratings of PFO shunt severity were documented in fewer than one in six TEE reports, underscoring the difficulties of its accurate assessment in routine clinical practice [51]. We adopted a pragmatic approach by grading shunt severity using a single still frame of the left heart with maximal contrast opacification, and applying a semiquantitative opacification scale. We propose this method for future studies employing high-density contrast agents.

### Limitations

Our study has several limitations. First, the relatively small sample size necessitates confirmation of these findings in larger studies. Second, the results are applicable only to a younger population, as all included patients were under 55 years of age. We administered a higher contrast volume (20 mL) instead of the usual 10 mL per injection, thus eliminating the need for a saline flush, and simplifying the procedure, but it may have resulted in greater cardiac opacification, potentially influencing the observed contrast grades. The arm side of the venous access for contrast administration was not recorded, and the inferior vena cava contrast injection was reported in 2 (3.7%) patients; however, these factors are unlikely to have affected the results. We also used different than usual approach to assess the left heart opacification grade as discussed. Given that the heart is a three-dimensional structure, any grading method based on a single imaging plane inherently underestimates the true left heart bubble volume [34]. Counting the number of bubbles in a 3D image is not currently feasible, but there are reports demonstrating better accuracy of 3D cTOE in distinguishing PFO and intrapulmonary shunt [52]. Despite every effort, minor discrepancies in Valsalva manoeuvre performance across centres may have occurred, particularly in patients undergoing cTOE under sedation, although this applied to < 20% of the study population and midazolam doses were low. Also, normal saline with added blood is the echocardiography contrast recommended as an alternative to normal saline for PFO assessment by American guidelines and used in many echo labs, although this approach is not universal [10, 53]. It would have been beneficial to compare results of the presented study using high density contrast and normal saline with blood; however, this was beyond the study scope. Finally, the inclusion of patients who demonstrated a right-to-left shunt on contrast echocardiography but had no invasively measurable shunt may have influenced the statistical relationship between bubble grade and shunt severity.

## Conclusion

Our study demonstrated that in studies using high-density contrast, cTOE and cTTE with Valsalva, but not rest studies, are highly sensitive for PFO assessment. There is no clinically relevant relationship between the left-heart contrast opacification grade and invasively measured right-to-left shunt severity or atrial septum/PFO morphology. The left-heart contrast opacification is equal to or higher in cTTE than in cTOE, especially in rest studies.

## Data Availability

The study datasets used and analysed are available from the corresponding author on reasonable request.
